# Heparanase gene deficiency suppresses atherosclerosis progression and enhances plaque stability in apolipoprotein E gene knockout mice with diabetes

**DOI:** 10.1042/CS20250817

**Published:** 2026-07-10

**Authors:** Tien K. Nguyen, Amy A. Baxter, Chloe Bourchier, Thanh Kha Phan, Enoch Chan, Ivan K.H. Poon, Shane R. Thomas, Mark D. Hulett

**Affiliations:** 1 Department of Biochemistry and Chemistry, La Trobe Institute for Molecular Science, La Trobe University, Melbourne, Victoria 3086, Australia; 2 Department of Pathology, School of Biomedical Sciences, Faculty of Medicine & Health, University of New South Wales, Sydney, New South Wales 2052, Australia

**Keywords:** atherosclerosis, diabetes, heparanase, inflammation

## Abstract

Diabetes mellitus accelerates the development of atherosclerotic cardiovascular disease. Heparanase, the sole enzyme responsible for cleaving heparan sulfates in the endothelial glycocalyx and subendothelial extracellular matrix, plays important pro-inflammatory roles in diabetes and atherosclerosis. However, the role of heparanase in diabetes-accelerated atherosclerosis is unknown. This study investigated the impact of *heparanase* gene-deficiency on atherosclerosis development in non-diabetic and streptozotocin (STZ)-treated, diabetic apolipoprotein E gene knockout (*ApoE^−/−^*) mice fed a normal chow diet for eight weeks. At the study endpoint, atherosclerotic lesion size and stability phenotype, along with blood glucose, serum lipid, and pro-inflammatory cytokine profiles were assessed. Compared to non-diabetic control *ApoE^−/−^* mice, STZ-treated, diabetic *ApoE^−/−^* mice exhibited significant increases in blood glucose, serum lipids, circulating MCP-1 and IL-6 levels, and the development of atherosclerotic lesion burden in the aortic sinus and aorta. While heparanase deficiency did not affect blood glucose or serum lipid levels, it significantly reduced the diabetes-dependent increase in circulating MCP-1 levels and diabetes-accelerated development of atherosclerotic lesions in the aortic sinus and aorta. Analysis of lesion composition and phenotype showed that diabetic *ApoE^−/−^* mice commonly exhibited advanced atherosclerotic lesions with well-developed necrotic cores, whereas diabetic heparanase-deficient *ApoE^−/−^* mice displayed early-stage atherosclerotic lesions, characterised by significantly smaller necrotic cores and increased collagen and smooth muscle cell content. The present study demonstrates that heparanase is a prominent pro-inflammatory protein driving the diabetes-accelerated formation of advanced atherosclerotic lesions with an unstable phenotype. Heparanase therefore represents a new therapeutic target for suppressing atherosclerotic cardiovascular disease in people with diabetes.

## Introduction

Diabetes is a chronic metabolic disorder characterised by elevated blood glucose levels (hyperglycaemia), which is recognised as a major cause of death globally. The prevalence of diabetes has risen dramatically with an estimated 537 million adults living with diabetes in 2021, compared to 108 million in 1980 (WHO global report on diabetes 2021). Alarmingly, the incidence of diabetes is predicted to increase to 783 million people by 2045 [1], highlighting the substantial and ongoing burden that diabetes poses for the global healthcare system.

It is well recognised that diabetes is associated with the accelerated development of atherosclerotic cardiovascular disease that increases clinical cardiovascular risk. As a result, individuals with diabetes exhibit a 2-to-4-fold greater risk of experiencing a clinical cardiovascular event compared to people without diabetes [2]. Accordingly, atherosclerotic cardiovascular disease is a major cause of mortality in people with diabetes, with 65% of all cardiovascular deaths occurring in individuals with diabetes [3]. The complex interaction of various key pathogenic factors is recognised to contribute to the accelerated development of atherosclerosis during diabetes including the development of hyperglycaemia and dyslipidaemia that heighten non-enzymatic glycation, oxidative stress, endothelial dysfunction, vascular inflammation, and the premature development of unstable or rupture-prone atherosclerotic lesions [4–6]. Roles of these key factors are also supported by studies in relevant animal models of diabetes-accelerated atherosclerosis, namely STZ-treated *ApoE^−/−^*mice [7–10]. Accordingly, great interest surrounds identifying the pro-inflammatory mediators and pathways augmenting atherosclerosis during diabetes. This study focused on the pro-inflammatory enzyme heparanase (Hpse).

Hpse is the sole enzyme capable of cleaving heparan sulfate (HS) side chains of HS-proteoglycans, a principal component of the vascular extracellular matrix (ECM) and basement membrane in mammalian cells [11]. Through HS cleavage at the endothelial glycocalyx and subendothelial layers, Hpse can promote vascular inflammation by altering endothelial permeability to circulating immune cells and lipoproteins [12].

Emerging evidence indicates Hpse contributes to the development of vulnerable atherosclerotic lesions. For example, Hpse expression is increased in human coronary lesions with the extent of increase highest in vulnerable coronary atherosclerotic plaques compared to stable plaques [13,14]. Also, our recent study established that Hpse gene deficiency suppressed atherosclerosis in *ApoE^−/−^* mice fed a Western-style high-fat diet [15].

In addition to atherosclerosis, Hpse is implicated in the development of diabetes. Thus, aortic endothelial cells secrete increased levels of Hpse in response to high glucose [16]. Expression levels of Hpse are also increased in patients with type 2 diabetes, with the extent of increase correlating with the degree of hyperglycaemia [17]. Hpse is also elevated in pancreatic β-cells and kidneys of diabetic mice, with *Hpse* gene-deficiency supressing the development of diabetic nephropathy [18,19]. Hpse-mediated HS cleavage also contributes to pancreatic β-cell destruction in mouse models of autoimmune type 1 diabetes [20].

While Hpse is implicated in the pathogenesis of both atherosclerosis and diabetes, no study has specifically addressed the role of Hpse in diabetes-accelerated atherosclerosis. This study addressed this unknown by studying the impact of *Hpse* gene-deficiency on atherosclerosis development in *ApoE^−/−^* mice rendered diabetic with STZ. We show that Hpse-deficient *ApoE^−/−^* mice exhibited a significant reduction in atherosclerosis burden and severity during diabetes, therefore identifying Hpse as a potential new therapeutic target to reduce atherosclerotic cardiovascular disease in people with diabetes.

## Materials and methods

### Mice

*ApoE^−/−^* mice on a C57Bl/6 background were obtained from The Jackson Laboratory (Bar Harbor, Maine, USA). *Hpse* gene-knockout (*Hpse*^−/−^) mice on a C57Bl/6 background were previously established by the targeted deletion of exon 1 of the mouse *Hpse* gene [21]. Mice deficient in both *ApoE* and *Hpse* genes were generated by crossbreeding *ApoE*^−/−^ mice with *Hpse*^−/−^ mice. Offspring heterozygous for both alleles were then crossed, and genotyping was performed to identify *ApoE*^−/−^ × *Hpse*^−/−^ double knockout mice, hereafter referred to as *AExH*^−/−^ mice. The successful establishment of this novel mouse strain was also validated by Western Blot and Hpse activity assays, as reported previously [15]. Colonies were maintained in the specific pathogen free facility at the La Trobe Animal Research and Teaching Facility. Experiments were carried out under the approval of the La Trobe University Animal Ethics Committee (AEC14-48) in accordance with Guidelines for Ethical Conduct in the Care and Use of Animals.

### Diabetes induction

Male *ApoE*^−/−^ and *AExH*^−/−^ mice at 8–12 weeks of age were randomly allocated to control or treatment groups. Male mice were exclusively used in this study, as female mice are known to be more resistant to STZ-induced diabetes [22]. For the induction of diabetes, the β-cell-selective toxin STZ (55 mg/kg in 0.1 M Na-citrate, pH 4.5; Sigma–Aldrich, St. Louis, Missouri, USA) was administered daily for 5 consecutive days by intraperitoneal (IP) injection. For control groups, 100 μl of citrate buffer (0.1 M Na-citrate, pH 4.5) vehicle was administered IP daily for 5 consecutive days. After the first injection, mice were provided 10% sucrose water for seven days, then replaced with standard water until the endpoint of the study. One week after the final STZ injection, blood was obtained via tail vein and blood glucose levels measuring using an Accu-Check glucose monitor (Roche, Basel, Switzerland). Mice presenting a blood glucose concentration at 15 mM or higher were considered diabetic [23] and studied alongside vehicle-control, non-diabetic mice for a further eight weeks and maintained on a normal chow diet. Over the eight-week study period, blood glucose was measured weekly, and body weight recorded twice weekly. At the study endpoint, mice were humanely killed by CO_2_ asphyxiation. Blood was immediately collected by cardiac puncture after euthanising. Relevant tissues were collected and fixed in 10% neutral buffered formalin for 24 h at room temperature for subsequent analyses.

### Serum analysis

Serum samples were collected from whole blood at the time of euthanasia, which was left at room temperature for 30 min for clotting. The clot was removed by centrifugation at 2,000 × ***g*** for 15 min at 4°C and the serum supernatant collected for analysis. For measurement of triglycerides, Infinity Triglycerides Reagent (Thermo Fisher Scientific) was added to 3 μl of undiluted serum or a calibration control (2 mM glycerol) and incubated at 37°C for 5 min before absorbance was measured at 500 nm. For measurement of total cholesterol, 50 μl of blood serum samples were added to a 96-well plate containing 50 μl of Amplex^®^ Red reagent containing 2 U/ml horseradish peroxidase, 2 U/ml cholesterol oxidase, and 0.2 U/ml cholesterol esterase (Amplex™ Red Cholesterol Assay Kit, Thermo Fisher Scientific). After 30 min incubation at 37°C, fluorescence intensity was measured using a SpectraMax M5e microplate reader with excitation at 545 nm and emission at 590 nm. Blood glucose measurements were determined from whole blood using an Accu-Check glucose monitor (Roche).

### Measurement of serum cytokines

Cytometric bead array (CBA) assay was used to measure the soluble levels of pro-inflammatory cytokines, MCP-1, TNF-α, and IL-6 in the mouse serum. Standard CBA flex sets were used to quantify circulating levels of MCP-1 and TNF-α, while an enhanced sensitivity flex set was used for IL-6. The CBA flex sets were obtained from BD Biosciences, and analyses performed as per the manufacturer’s instructions. Samples were analysed using a BD FACS Canto II flow cytometer and BD FACS Diva software and data were analysed using BD CBA FCAP array software.

### Tissue processing, histological analysis, and atherosclerotic lesion classification

Animals were humanely killed by CO_2_ asphyxiation and blood was collected immediately by cardiac puncture. Following PBS perfusion, the heart and the entire aorta including the aortic arch, thoracic, and abdominal aorta were excised and fixed in 10% neutral buffered formalin for at least 24 h at room temperature.

The whole aorta was prepared for *en face* analysis of atherosclerotic lesion burden by Oil-red-O staining of aortic lesion lipids present. Images were captured using an Olympus SZ61 Stereomicroscope (Minato, Tokyo, Japan) with a digital camera (Minato, Tokyo, Japan). The aortic surface area containing Oil-red-O-stained atherosclerotic lesions was measured using ImageJ software (National Institutes of Health Imaging; https://imagej.nih.gov/ij) and expressed as a percentage of the total aortic surface area positive for Oil-red-O staining.

For analysis of aortic sinus atherosclerosis lesion size and stability phenotype, 5 μm proximal heart tissue sections were stained with Mayer’s haematoxylin/eosin (H&E) to measure atherosclerotic lesion and necrotic core size or stained with Picrosirius red/fast green (PSR) to detect collagen. To quantify atherosclerotic lesion areas, two to four different positions of the aortic sinus were analysed, each separated by 80–100 μm, with the most proximal site commencing after the appearance of two aortic valve leaflets. The relevant H&E-stained aortic sinus sections were imaged using an Olympus BX41 microscope and the area of lesions within the valve leaflets of the aortic sinus measured using ImageJ and expressed as lesion area (μm^2^). Data were obtained from the average of 2–4 sections per mouse. In the same tissue sections, necrotic core size was measured as the lesion area devoid of nucleated cellular tissue and expressed as a percentage of the total lesion area. A Zeiss Axioscan 7-slide scanner was used to image PSR-stained sections. Lesion collagen content was measured as the PSR-positive area within the atherosclerotic lesions, quantified manually by ImageJ and expressed as a percentage of the total lesion area.

Atherosclerotic lesions were categorised for lesion severity based on previously described criteria [24] and classified as fatty streaks, mild lesions, moderate lesions, and severe lesions. Data were obtained from the average of 2–4 sections per mouse and lesion types were expressed as a percentage of total lesion types.

### Immunohistochemical staining

Slides containing 5 μm paraffin-embedded tissue sections were dewaxed and endogenous peroxidase activity was blocked by incubating the sections in 3% hydrogen peroxide in 100% methanol for 30 min at room temperature. Slides were then blocked with blocking buffer containing 3% normal goat serum (Vector Laboratories, Burlingame, Ca, USA) diluted in PBS/Tween (0.1%) for 1 h at room temperature in a humidified chamber before being incubated with primary antibodies anti-Mac3 (Ab13524, 2 μg/ml, Abcam), anti-CD68 (Ab 125212, 1 μg/ml, Abcam), anti-MCP-1 (Ab 7202, 5 μg/ml, Abcam), anti-CD31 (Ab 28364, 0.5 μg/ml, Abcam), anti-VCAM-1 (Ab 134047, 2.5 μg/ml, Abcam), or anti-SMA (Ab21027, 1 μg/ml, Abcam) at 4°C for overnight in a humidified chamber. Sections were washed and incubated with biotinylated goat anti-rat, goat anti-rabbit, or anti-goat secondary antibody (Vector Laboratories) for 1 h at room temperature. Slides were then incubated with Vectastain avidin–biotin complex (Vector Laboratories) at room temperature for 30 min. Staining was visualised with Vector DAB (3,3′-diaminobenzidine) substrate (Vector Laboratories) and sections were counterstained with hematoxylin. Negative controls were obtained by incubating with secondary antibody only. Images were captured using a Zeiss Axioscan 7 slide scanner. Expression of targeting antigens was quantified using ImageJ software and defined as a percentage of the total lesion area.

### Statistical analysis

The investigators were blinded to group allocation during data collection and analysis. Statistical analysis was performed using GraphPad Prism, version 8.1.1 (GraphPad Software). Normality testing was performed using Anderson-Darling and D’Agostino and Pearson normality tests in GraphPad Prism. Comparison between two groups was performed using a Student’s unpaired *t*-test for parametric data and Mann–Whitney test for nonparametric data. For multiple comparisons, one-way analysis of variance (ANOVA) with Bonferroni post hoc tests were performed for data with normal distribution or the Kruskal–Wallis test with Dunn’s multiple comparisons post hoc test were used for non-normal distribution. The experimental numbers (*n*) are listed in the figure legends. *n* represents the number of biological replicates. All data are expressed as the mean ± SEM and *P-*values less than 0.05 were considered statistically significant.

## Results

### *Hpse* gene deficiency does not affect changes to body weight, blood glucose, and lipid profiles in STZ-treated ApoE^−/−^ mice with diabetes

To investigate the impact of Hpse on diabetes-accelerated atherosclerosis, we treated ApoE^−/−^ and *AExH*^−/−^ mice with STZ, which induces the destruction of pancreatic β-cells and resultant increase in blood glucose that augments atherogenesis in these mice [25,26]. After diabetes induction with STZ, control and diabetic ApoE^−/−^ and *AExH*^−/−^ mice were fed a normal chow diet for 8 weeks after which the extent of atherosclerosis was analysed. As expected, STZ-treated *ApoE*^−/−^ mice showed a significant increase in Hpse protein expression levels, including both the inactive (65 kDa) and active (50 kDa) forms compared with heathy control *ApoE^−/−^* mice (Supplementary Figure S1).

**Figure 1:**
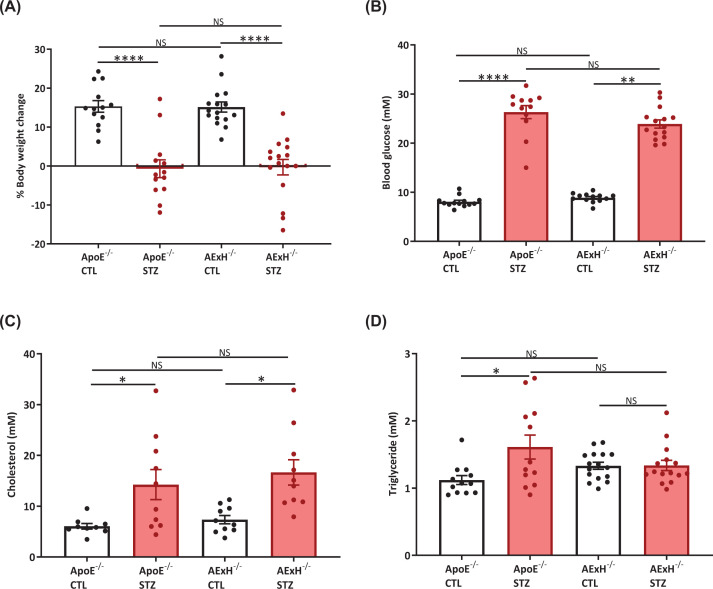
Hpse deficiency does not affect changes in body weight, blood glucose and serum lipid levels in control or diabetic *ApoE*^−/−^ and *AExH*^−/−^ mice (**A**) Percentage of body weight change and levels of (**B**) blood glucose (mM), (**C**) serum cholesterol (mM), and (**D**) serum triglyceride (mM) in non-diabetic/control (CTL) and STZ-treated/diabetic (STZ) *ApoE*^−/−^ and *AExH*^−/−^ mice at 8-weeks post-diabetes induction. Data represent the mean ± SEM (*n* = 9–16). Statistically significant differences determined by a one-way ANOVA, followed by Bonferroni multiple comparisons test or the Kruskal–Wallis test with Dunn’s multiple comparisons post hoc test (NS, not significant; *P<0.05, **P<0.01, ***P<0.001, ****P<0.0001).

Over the 8-week period both *ApoE*^−/−^ or *AExH*^−/−^ control mouse groups similarly gained approximately 15% in body weight. The mean body weight of STZ-treated *ApoE*^−/−^ or *AExH*^−/−^ mouse groups remained unchanged over the 8-week period consistent with the onset of diabetes that inhibits ongoing weight gain (Figure 1A). While blood glucose levels of control *ApoE*^−/−^ and *AExH*^−/−^ mice remained steady at approximately 10 mM, STZ-treated *ApoE*^−/−^ and *AExH*^−/−^ mice exhibited increased mean blood glucose levels of 26 and 24 mM, respectively, indicative of the establishment of diabetes in both mouse genotypes. The extent of increase in blood glucose levels was not different between STZ-treated diabetic *ApoE*^−/−^ and *AExH*^−/−^ mice (Figure 1B). Compared to non-diabetic control mice, total cholesterol levels were also significantly elevated in STZ-treated diabetic *ApoE*^−/−^ and *AExH*^−/−^ mouse groups, although no significant difference between the mouse genotypes was observed (Figure 1C). No significant difference in serum triglyceride levels was noted between STZ-treated diabetic *ApoE*^−/−^ and *AExH*^−/−^ mice, although a significant increase was observed in diabetic *ApoE*^−/−^ compared to non-diabetic control *ApoE*^−/−^ mice (Figure 1D). Taken together, these findings indicate that *Hpse* gene deficiency does not affect diabetes-dependent changes in body weight, blood glucose, cholesterol, and triglyceride levels in *ApoE*^−/−^ mice.

### *Hpse* gene-deficient *ApoE*^−/−^ mice exhibit reduced atherosclerotic lesion development during diabetes

**Figure 2:**
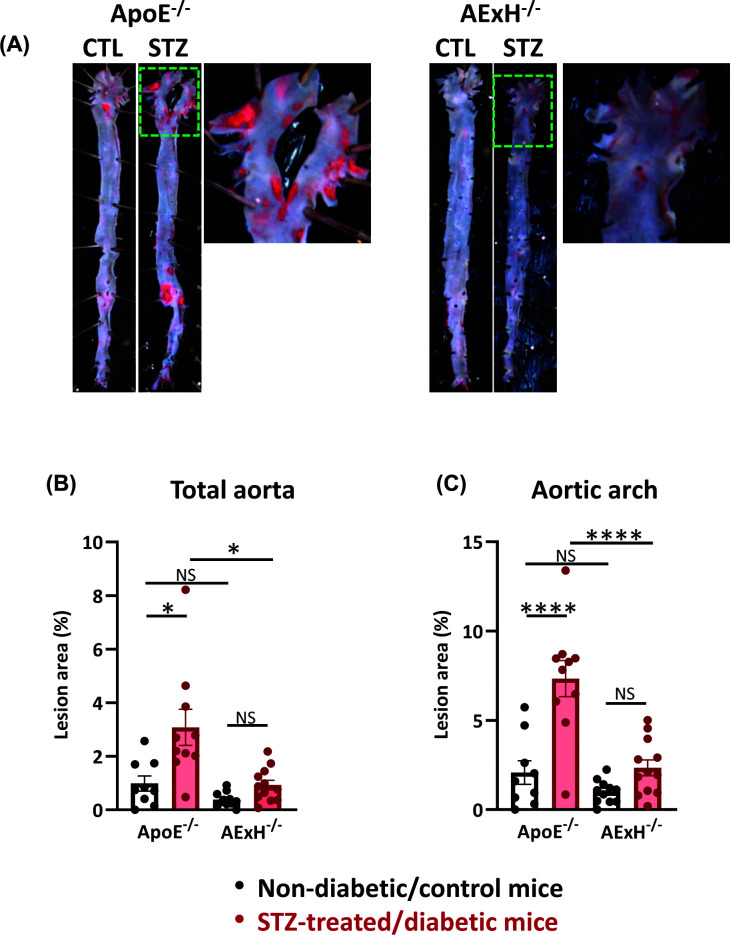
Hpse deficiency inhibits the diabetes-dependent increase in atherosclerotic burden in the aorta of *ApoE*^−/−^ mice (**A**) Representative images of Oil-red-O-stained aortas from non-diabetic/control (CTL) and STZ-treated/diabetic (STZ) *ApoE*^−/−^ and *AExH*^−/−^ mice at 8-weeks post-diabetes induction. Quantification of the aortic surface area positively stained for Oil-red-O in (**B**) total aorta and (**C**) aortic arch of mice. Data represent the mean ± SEM (*n* = 9–12). Statistically significant differences determined by a one-way ANOVA, followed by Bonferroni multiple comparisons test or the Kruskal–Wallis test with Dunn’s multiple comparisons post hoc test (NS, not significant; *P<0.05, ****P<0.0001).

**Figure 3:**
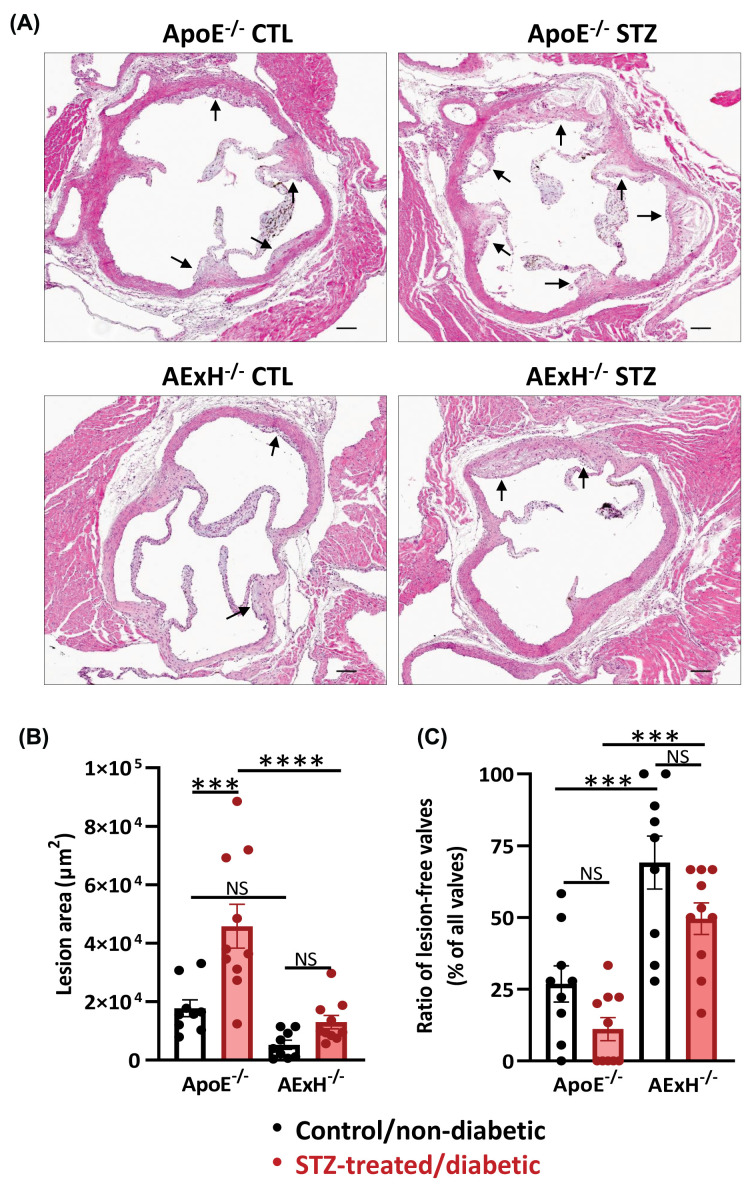
Hpse-deficiency reduces atherosclerotic burden in the aortic sinus of diabetic *ApoE*^−/−^ mice (**A**) Representative H&E-stained aortic sinus sections of non-diabetic/control (CTL) and STZ-treated/diabetic (STZ) *ApoE*^−/−^ and *AExH*^−/−^ mice at 8-weeks post-diabetes induction. Quantification of (**B**) atherosclerotic plaque size μm^2^ and (**C**) % of lesion-free valves in the aortic sinus. Scale bars indicate 100 μm. Data represent the mean ± SEM (*n* = 9–10). Statistically significant differences determined by a one-way ANOVA, followed by Bonferroni’s multiple comparisons test (NS, not significant; ***P<0.001, ****P<0.0001).

We next investigated whether Hpse deficiency impacts on atherosclerotic lesion development in *ApoE*^−/−^ mice during diabetes. At the experimental endpoint, the degree of atherosclerosis was analysed in aortas by *en face* analysis of Oil-red-O-stained lipids (Figure 2A) and in the aortic sinus as lesion area measured by morphometry (Figure 3A). *En face* analysis showed that compared to non-diabetic control *ApoE*^−/−^ mice, STZ-treated diabetic *ApoE*^−/−^ mice exhibited a significant increase in atherosclerotic lesion development in the total aorta (Figure 2B) and aortic arch (Figure 2C). This diabetes-dependent increase in aortic atherosclerosis was, however, significantly inhibited in diabetic *AExH^−/−^* mice, with the degree of reduction most apparent in the aortic arch where the development of aortic atherosclerosis is most prominent (Figure 2C) [27]. No difference in aortic lesion development was apparent between non-diabetic *ApoE*^−/−^ and *AExH*^−/−^ mice and between STZ-treated diabetic and control non-diabetic *AExH*^−/−^ mice.

Morphometric analysis of H&E-stained aortic sinus sections also showed that diabetic *ApoE*^−/−^ mice exhibited a significant increase in lesion development compared to control *ApoE*^−/−^ mice (Figure 3B). Moreover, a significant decrease in atherosclerotic lesion burden was apparent in STZ-treated diabetic *AExH*^−/−^ mice compared to STZ-treated diabetic *ApoE*^−/−^ mice (Figure 3B). No significant difference in aortic sinus atherosclerosis was observed between diabetic and non-diabetic *AExH*^−/−^ mice (Figure 3B).

The incidence of lesion-free aortic sinus valves was next enumerated. For both non-diabetic and diabetic conditions, *AExH*^−/−^ mice showed a significantly higher proportion of lesion-free valves when compared to the *ApoE*^−/−^ mouse counterparts. Thus, in non-diabetic and diabetic *AExH*^−/−^ mice approximately 70% and 50%, respectively of all aortic sinus valves were free of lesions compared to non-diabetic and diabetic *ApoE*^−/−^ mice in which approximately 25% and 10% of valves, respectively were lesion free (Figure 3C). Collectively, these findings indicate that *Hpse* gene deficiency significantly suppresses the diabetes-accelerated development of atherosclerotic lesions in *ApoE*^−/−^ mice.

### *Hpse* gene deficiency promotes atherosclerotic lesion stability during diabetes

**Figure 4:**
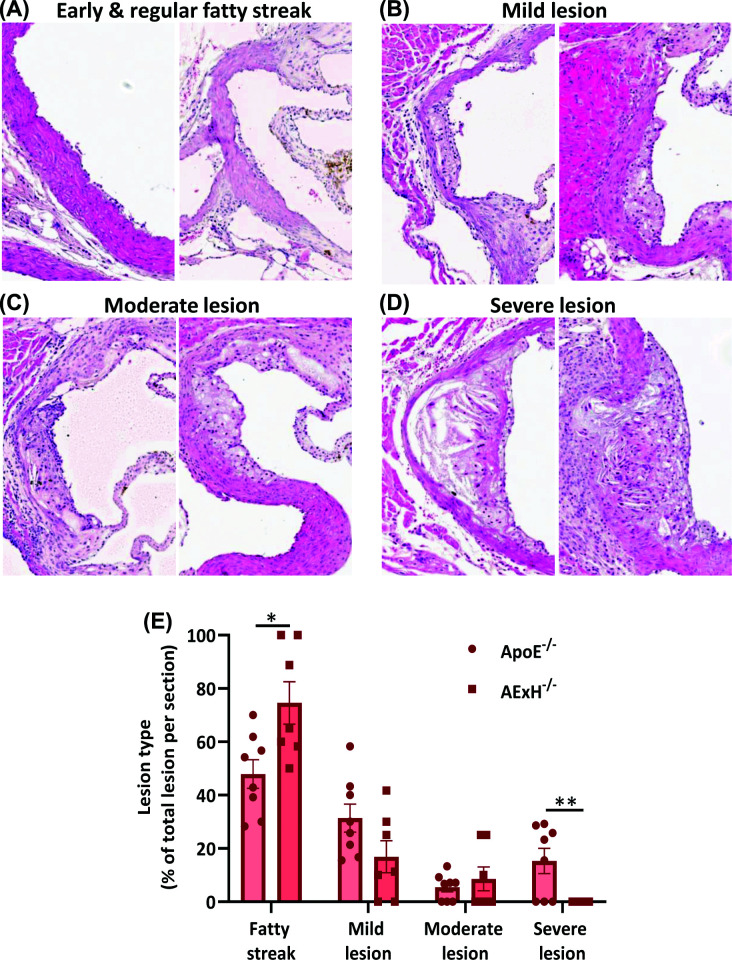
Classification of atherosclerotic lesion severity in diabetic *ApoE*^−/−^ and *AExH*^−/−^ mice (A–D) Representative images of different atherosclerotic lesion types in H&E-stained aortic sinus tissue from STZ-treated, diabetic *ApoE*^−/−^ mice categorized as: (**A**) early or regular fatty streak (type I or II) lesions that contain a maximum of 10 or >10 foam cells within the intima per lesion cross-section, respectively, (**B**) mild (type III) lesions where foam cells extend into media and/or lesions are covered by a fibrous cap, (**C**) moderate or intermediate (type IV) lesions where the developing lesion has advanced or infiltrated into the media, and exhibits fibrosis without architectural loss, and (**D**) severe (type V) lesions in which the media is severely damaged, elastic lamina is broken, and cholesterol crystals and/or necrosis are present [24]. (**E**) Quantification and comparison of atherosclerotic lesion type in diabetic *ApoE*^−/−^ and *AExH*^−/−^ mice. Data represent the mean ± SEM (*n* = 7–8). Statistically significant differences determined by multiple *t*-test, followed by the Holm–Sidak method (*P<0.05, **P<0.01).

We next examined the impact of Hpse on atherosclerotic lesion severity and stability in aortic sinus lesions. Atherosclerotic lesions were classified into early/regular fatty streak (Figure 4A), mild (Figure 4B), moderate (Figure 4C), or advanced/severe (Figure 4D) atherosclerotic lesions, as described previously [24]. Diabetic STZ-treated *ApoE*^−/−^ mice exhibited all lesion types with fatty streak, mild, moderate, and severe lesions accounting for 48%, 31%, 5%, and 15%, respectively (Figure 4E). Compared to diabetic *ApoE*^−/−^ mice, diabetic *AExH*^−/−^ mice exhibited a marked increase in the prevalence of early fatty streak lesions to 75%, with mild and moderate lesions accounting for remaining 17% and 9% of lesions (Figure 4E). Notably no severe lesions were detected in diabetic *AExH*^−/−^ mice (Figure 4E).

Development of different lesion types was also examined and compared between non-diabetic and diabetic mice of both mouse strains (Supplementary Figure S2). Notably, the trend for atherosclerotic lesion development in diabetic *AExH*^−/−^ mice resembles that of non-diabetic *ApoE*^−/−^ mice (Supplementary Figure S2E).

The high proportion of lesion-free valves observed in non-diabetic *AExH*^−/−^ control mice, where approximately 70% of mice showed no detectable lesions within the aortic sinus (Figure 3F), limited meaningful comparison with other experimental mouse groups. Therefore, only aortic sinus cross-sections of diabetic *ApoE^−/−^* and *AExH^−/−^* mice were employed for subsequent immunohistochemical (IHC) analysis.

**Figure 5:**
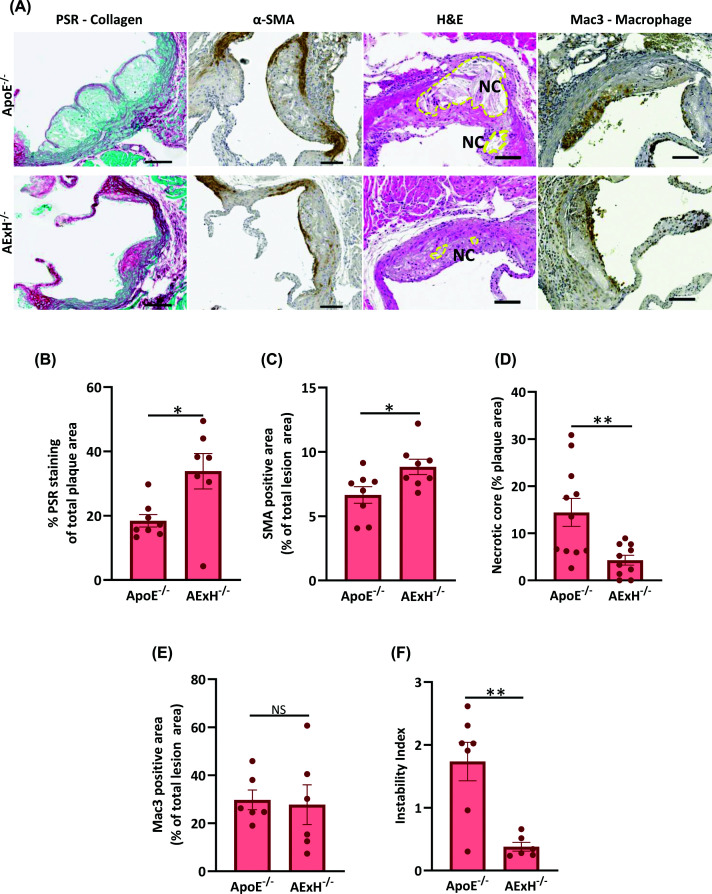
Hpse deficiency increases collagen content and reduces necrotic core development in atherosclerotic lesions from *ApoE*^−/−^ mice with diabetes (**A**) Representative images of aortic sinus atherosclerotic lesions from STZ-treated, diabetic *ApoE*^−/−^ and *AExH*^−/−^ mice analysed for collagen content by PSR-staining, SMC content by IHC-staining for α-SMA, necrotic core by H&E staining, and macrophage content by IHC-staining for MAC-3 aortic sinus sections. Quantification of (**B**) collagen content, (**C**) SMC (α-SMA) content, (**D**) necrotic core size, and (**E**) macrophage content within aortic sinus atherosclerotic plaques of aortic sinus sections from diabetic *ApoE*^−/−^ and *AExH*^−/−^ mice. (**F**) Quantification of plaque instability index defined as the sum of unstable lesion markers (i.e. % necrotic core plus % macrophage content) versus the sum of stable lesion markers (i.e. % collagen plus % SMC content). NC: necrotic core. Scale bars indicate 100 μm. Data represent the mean ± SEM [*n* = 6–8 for panels (B), (C), (E), and (F); *n* = 10–11 for (D)]. Statistically significant differences determined by two-tailed Mann–Whitney test or Student unpaired, two-tailed *t*-test (NS: not significant, *P<0.05, **P<0.01).

Next, the expression of CD31 and VCAM-1, markers of endothelial cell integrity and activation, respectively, was examined by IHC. No significant differences in the expression of either CD31 or VCAM-1 was observed between diabetic *AExH*^−/−^ mice and diabetic *ApoE*^−/−^ mice (Supplementary Figure S3). We then assessed atherosclerotic lesion stability phenotype by measuring lesion content of collagen and smooth muscle cells (SMCs) relative to lesion necrotic core size and macrophage content. For collagen and SMC, compared to diabetic *ApoE*^−/−^ mice, diabetic *AExH*^−/−^ mice showed significant increases in lesion content of collagen (Figure 5B) and SMCs (Figure 5C). For necrotic core and macrophages, diabetic *AExH*^−/−^ mouse lesions exhibited a significant reduction in the necrotic core size (Figure 5D) and no significant change in macrophage content (Figure 5E and Supplementary Figure S4) compared to lesion from *ApoE*^−/−^ mice with diabetes. When indexing lesion instability as a function of unstable markers (i.e. necrotic core and macrophage content) versus stable markers (i.e. collagen and SMC content), diabetic *AExH*^−/−^ mice exhibited a significant reduction compared to diabetic *ApoE*^−/−^ mice (Figure 5F). Taken together, these data further indicate that Hpse is a critical factor promoting atherosclerotic lesion severity and instability during diabetes.

### *Hpse* gene deficiency reduces the levels of circulating MCP-1 during diabetes

**Figure 6:**
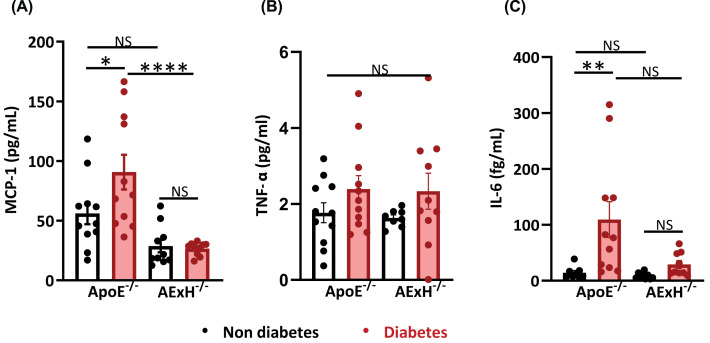
Hpse-deficiency inhibits the diabetes-induced increase in circulating MCP-1 levels in *ApoE*^−/−^ Circulating levels of (**A**) MCP-1, (**B**) TNF-α, and (**C**) IL-6, were determined by CBA assay. Data represent the mean ± SEM (*n* = 9–11). Statistically significant differences determined by a one-way ANOVA, followed by Bonferroni multiple comparisons test or the Kruskal–Wallis test with Dunn’s multiple comparisons post hoc test (NS, not significant; *P<0.05, **P<0.01, ****P<0.0001).

Protein levels of proinflammatory cytokines TNF-α, IL-6, MCP-1 were next measured (Figure 6). Among cytokines, circulating MCP-1 and IL-6 levels were significantly increased in STZ-treated diabetic *ApoE*^−/−^ mice compared to non-diabetic *ApoE*^−/−^ mice. Notably, this diabetes-dependent increase in circulating MCP-1 was significantly inhibited in *AExH*^−/−^ mice (Figure 6A), although no difference in the local expression of MCP-1 within atherosclerotic lesions was apparent between diabetic *AExH*^−/−^ and *ApoE*^−/−^ mice (Supplementary Figure S5). A non-significant trend for reduced IL-6 was also apparent in diabetic *AExH*^−/−^ mice compared to diabetic *ApoE*^−/−^ mice (Figure 6C). No significant changes in circulating TNF-α levels were observed (Figure 6B).

## Discussion

Although Hpse is implicated in the pathogenesis of both type 1 diabetes, and atherosclerosis and atherosclerotic lesions of diabetic, hyperlipidemic swine show increased Hpse expression [28], the role of Hpse in diabetes-accelerated atherosclerosis is unknown. To address this, we studied the impact of *Hpse* gene deficiency on the development of atherosclerosis in *ApoE*^−/−^ mice rendered diabetic with STZ. *Hpse* gene deficiency suppressed the diabetes-accelerated increase in atherosclerotic lesion burden at the aortic sinus, aortic arch, and descending aorta in diabetic *ApoE*^−/−^ mice independent of differences in blood glucose and lipid levels. Moreover, Hpse deficiency reduced the severity and instability phenotype of the atherosclerotic lesions formed in diabetic *ApoE*^−/−^ mice. Additionally, Hpse deficiency inhibited the diabetes-induced increase in circulating levels of the pro-inflammatory cytokine MCP-1. Therefore, this study identifies Hpse as critical pro-inflammatory protein driving the accelerated development of advanced atherosclerotic lesions with an unstable phenotype during diabetes.

During diabetes, hyperglycaemia and dyslipidaemia are key risk factors that promote damage and dysfunction to the vasculature to augment the development, severity, and clinical risk of atherosclerotic cardiovascular disease [29,30]. The current study showed that induction of diabetes in *ApoE^−/−^* mice resulted in the onset of hyperglycaemia and dyslipidaemia, as indicated by significant increases in blood glucose and serum cholesterol and triglyceride levels, which paralleled the accelerated development of atherosclerosis. The onset of STZ-induced diabetes also retarded body weight gain in *ApoE^−/−^* mice, consistent with prior studies [25,31,32]. While pharmacological inhibition of Hpse with high-dose PG545 has been reported to reduce blood glucose levels in *ApoE*^−/−^ mice that were fed normal chow [33], treatment of high-fat-diet (HFD)-induced atherosclerotic *ApoE*^−/−^ mice with either low or high doses of PG545 did not alter the blood glucose levels [34]. In the current study, although Hpse deficiency suppressed the development and severity of atherosclerosis in diabetic *ApoE^−/−^* mice, the Hpse-dependent inhibition of atherogenesis occurred independently of the diabetes-induced changes to blood glucose, plasma lipids, and body weight. Consistent with our findings, previous studies in STZ-treated diabetic C57Bl/6 mice or HFD-fed *ApoE^−/−^* mice have also reported no impact of Hpse deficiency on blood glucose or lipid levels [15,18], indicating that Hpse promotes diabetes-accelerated atherogenesis by augmenting other pro-atherogenic processes.

In the current study, STZ-induced diabetic *ApoE*^−/−^ mice deficient in the *Hpse* gene exhibited a significant reduction in atherosclerotic lesion burden, accompanied by a significant increase in the proportion of lesion-free valves and a higher prevalence of immature fatty streak lesions, with no development of severe lesions in the aortic sinus. These data suggest that Hpse deficiency plays an athero-protective role by delaying atherosclerotic lesion development and progression during diabetes. This notion is further supported by the findings from our recent study in which *Hpse*-deficient *ApoE^−/−^* mice fed a Western-style HFD exhibited significantly reduced atherosclerotic lesion burden in the aorta and aortic sinus [15].

Considering Hpse is a prominent pro-inflammatory enzyme capable of promoting vascular inflammation by facilitating immune cell infiltration, retention, and activation in the vascular wall [11,13,35], it is plausible that Hpse accelerates atherogenesis during diabetes via its impact on inflammatory processes. Indeed, Hpse is involved in Toll-like receptor (TLR) and NF-κB signalling pathways, whereby its enzymatic activity generates soluble HS fragments that activate TLR and NF-κB pathways, leading to the release of proinflammatory cytokines such as MCP-1, TNF-α, IL-6, and IL-1β [36]. The involvement of Hpse in TLR signalling is further supported by findings that Hpse-induced TNF-α production is attenuated by inhibition of MyD88, a key adaptor in TLR pathways, as well as by neutralising antibodies against TLR2 and TLR4 [13].

Consistent with these observations, Hpse deficiency suppressed the diabetes-dependent increase in circulating levels of the pro-inflammatory and pro-atherogenic chemokine MCP-1 [37–39], the levels of which are increased during diabetes [40]. Hpse deficiency also reduced circulating MCP-1 levels in HFD-fed *ApoE*^−/−^ mice [15] supporting that Hpse is a key regulator of MCP-1 during atherogenesis in response to both hypercholesterolaemia and diabetes. Notably, MCP-1 is also a well-characterised HS-binding chemokine such that HS-cleavage by Hpse represents a plausible mechanism facilitating the release of MCP-1 chemokine reservoirs in the vascular glycocalyx and sub-endothelial ECM [41,42]. Elevated levels of MCP-1 are considered to promote atherogenesis via the chemotactic recruitment and accumulation of activated leukocytes, namely monocyte-derived macrophages, into the arterial wall [37–39]. Therefore, the reduction in MCP-1 may represent one mechanism by which Hpse deficiency protects against diabetes-accelerated atherosclerosis.

Within atherosclerotic lesion, MCP-1 is primarily expressed by endothelial cells, SMCs, and macrophages [43]. Notably, MCP-1 has been reported to be highly expressed in the macrophage-rich area of the atherosclerotic lesions in human and animal models [44]. In the present study, no apparent difference in the local expression of MCP-1 levels in atherosclerotic lesions was observed between diabetic *ApoE*^−/−^ mice and Hpse-deficient diabetic *ApoE*^−/−^ mice, when expressed as a function of lesion area. This finding is consistent with no differences in macrophage and endothelial cell content within atherosclerotic lesions between these mouse groups, despite the increased SMC content observed in Hpse-deficient diabetic *ApoE*^−/−^ mice. These results suggest that Hpse deficiency may not significantly alter local MCP-1 production within the plaque. However, measuring MCP-1 via IHC and as a function of lesion area does not necessarily reflect MCP-1 bioactivity and hence more detailed studies are required to define the role of Hpse in controlling the local inflammatory responses controlled by MCP-1 and other pro- and anti-inflammatory cytokines within atherosclerotic lesions under diabetic conditions.

An important event for atherosclerotic lesion formation and development is endothelial cell dysfunction and the subsequent recruitment of immune cells into the subendothelial space of the artery [4,45]. Both MCP-1 and VCAM-1 are expressed by activated endothelium [46,47]. However, in the present study, no significant differences were observed in CD31 expression, nor in MCP-1 or VCAM-1 levels within atherosclerotic lesions, between diabetic *ApoE*^−/−^ mice and Hpse-deficient diabetic *ApoE*^−/−^ mice, when expressed as a function of lesion area. These findings suggest that the pro-atherogenic effects of Hpse during diabetes may occur independently of changes in endothelial cell activation. Nevertheless, further *in vivo* and *in vitro* studies are required to determine whether Hpse influences endothelial activation and function under diabetic conditions.

Hpse has been shown to increase the numbers of CD45^+^ leukocytes into atherosclerotic aorta from *ApoE^−/−^* mice fed a HFD [15]. Additionally, Hpse is implicated in facilitating the infiltration of different immune cell subsets into inflamed tissues in different disease settings including macrophages [48], monocytes [35], neutrophils [35], dendritic cells [21] and T cells [19]. Therefore, the pivotal role of Hpse in promoting atherosclerotic lesion development during diabetes likely involves the enzymes capacity to modulate the recruitment of immune cells into the intimal space. Further detailed immune profiling investigations are therefore warranted to determine the extent to which the pro-atherogenic action of Hpse during diabetes reflect alterations in the recruitment, retention and functional phenotypes of different immune cell sub-sets in atherosclerotic lesions.

During diabetes, atherosclerotic cardiovascular disease manifests clinically due to the formation of vulnerable or unstable advanced atherosclerotic plaques that are at risk of rupture and subsequent formation of an occlusive arterial thrombus that can lead to myocardial infarction. The stability of atherosclerotic lesions is dictated by various key factors including the prevalence of vascular SMC and integrity/thickness of the collagen-rich fibrous cap versus the content of pro-inflammatory macrophages and size of the dangerous acellular necrotic core [49]. In the present work, we observed that lesions of Hpse-deficient diabetic *ApoE*^−/−^ mice exhibited a significant reduction in necrotic core size in parallel with significant increases in collagen and SMC content. However, there was no significant changes in the content of lesion macrophages when expressed as a percentage of total lesion area. Accordingly, when lesion instability was indexed as a function of macrophage content and necrotic core size versus SMC content and collagen content, Hpse deficiency afforded the formation of lesions in diabetic *ApoE*^−/−^ mice with a significantly more stable phenotype. Therefore, these findings on atherosclerotic lesion composition and phenotype support a critical role of Hpse in promoting not only the development of atherosclerotic lesions during diabetes but also plaque instability. These observations are consistent with the findings from our recent study reporting that Hpse-deficient *ApoE*^−/−^ mice fed a Western-style HFD for 14 weeks afforded the formation of advanced lesions with a reduced necrotic core size and increased SMC content [15]. Together, the findings of the current and prior work indicate Hpse is an important determinant of atherosclerotic lesion burden and stability in response to diabetes or hyperlipidaemia.

Hpse is reported to play a significant role for controlling the recruitment of monocytes [50] and macrophages [51] into tissue sites of inflammation. Although Hpse deficiency reduced atherosclerosis burden, severity and lesion instability phenotype in diabetic *ApoE^−/−^* mice, no significant difference in the content of lesion macrophages was noted between diabetic *ApoE^−/−^* and *AExH^−/−^* mice when expressed as a function of lesion area. This is similar to our prior data in high-fat-fed *ApoE^−/−^* mice where Hpse deficiency did not impact macrophage content as a function of lesion area despite inhibiting atherogenesis [15]. Hpse may, however, impact macrophage activation status and phenotype. Thus, Hpse can directly activate macrophages, elevating the expression of pro-inflammatory cytokines MCP-1 and IL-1β [31]. Therefore, further studies are warranted into the role of Hpse in controlling lesion monocyte and macrophage function and phenotype during diabetes-accelerated atherosclerosis. Such studies will require the application of advanced approaches such as single-cell RNA sequencing, multi-parameter flow cytometry of digested aortic tissues, and/or spatial transcriptomics, which enable high-resolution characterisation of immune cell subsets, activation states, and functional pathways within the atherosclerotic lesion microenvironment [52].

The formation of the necrotic core is a hallmark of advanced atherosclerotic plaques and represents an early indicator of plaque instability. In advanced atherosclerotic plaques, the efficiency of phagocytes to clear apoptotic and necrotic materials, a process known as efferocytosis, becomes impaired [53]. This defective efferocytosis results in the accumulation of an acellular pro-inflammatory and pro-thrombotic necrotic core, which is enriched with extracellular cholesterol crystals and cell debris derived from dying foam cells and other apoptotic/necrotic lesion cells [54,55]. Importantly, Hpse has been shown to induce cell death via necroptosis [56]. Therefore, the significant reduction in the necrotic core development observed in the atherosclerotic lesions of diabetic Hpse-deficient mice in this study could be related to the capacity of Hpse to promote lesion cell death. More detailed studies into the impact of Hpse on cell death mechanisms in atherosclerosis are therefore warranted.

Matrix metalloproteinases (MMPs) are well-recognised for their role in degrading ECM components of the fibrous cap, leading to structural weakening and plaque instability [57]. Co-localisation of MMPs with regions of degraded ECM in vulnerable plaques has also been demonstrated [58]. In particular, MMP-9 is predominantly localised to the shoulder regions, necrotic core, and fibrous cap of atherosclerotic plaques, with increased expression and activity observed in unstable compared to stable plaques [58, 59]. In the present study, however, no significant differences in MMP9 expression were observed between diabetic *ApoE^−/−^* mice and Hpse-deficient diabetic *ApoE^−/−^* mice when expressed as a function of lesion area (Supplementary Figure S6), suggesting that the stabilising effects of Hpse deficiency may occur independently of changes in MMP-9 expression. Notably, Hpse has been reported to act in concert with proteases such as MMPs and PCSK6 to promote fibrous cap degradation and plaque instability [60]. Furthermore, a marked elevation of MMPs such as MMP-2 and MMP-14, was observed in Hpse-deficient mice, suggesting that MMPs provide tissue-specific compensation for the lack of Hpse expression [61]. Therefore, further investigation is warranted to determine whether the expression and activity of other MMPs as well as tissue inhibitor of metalloproteinases within atherosclerotic plaques are altered in the absence of Hpse.

Plaque calcification is another hallmark of advanced atherosclerosis. Notably, Hpse has been implicated in the regulation of vascular calcification. While Hpse has been shown to promote calcification of human vascular SMCs *in vitro*, its expression is correlated with genes associated with osteoclast differentiation and function in human atherosclerotic lesions. Furthermore, Hpse is expressed by osteoclast-like cells within human carotid atherosclerotic lesions [62]. These findings indicate that Hpse not only contributes to plaque progression and instability but may also play a role in vascular calcification, a key feature of advanced atherosclerotic lesions.

Although the current study identifies Hpse as a critical enzyme driving diabetes-accelerated atherogenesis in *ApoE^−/−^* mice, a limitation is that male mice were exclusively studied. This is because the development and severity of atherosclerosis in mouse models of disease and human cardiovascular disease patients are influenced by gender [63]. Indeed, female mice are well-documented to be more resistant to STZ-induced diabetes [22]. As a result, male mice were exclusively used in this study to minimise variability from hormonal fluctuations, and to allow us to obtain a clear assessment of Hpse in this disease setting. However, it will be interesting to perform future studies in both male and female diabetic *ApoE^−/−^* and *AExH^−/−^* mice to identify any gender-specific differences in the impact of Hpse on diabetes-accelerated atherogenesis.

It should be noted that the present study specifically investigated the role of Hpse in a model of type 1 diabetes-accelerated atherosclerosis, which was induced by treating atherosclerosis-prone *ApoE^−/−^* mice with multiple low-doses of STZ (55 mg/kg/day for 5 consecutive days). However, to comprehensively define the role of Hpse in diabetes-accelerated atherosclerosis, future studies should also investigate the role of Hpse in atherosclerosis under the setting of type 2 diabetes and metabolic syndrome. In addition, although severe atherosclerotic lesions were observed in diabetic *ApoE^−/−^* mice, the mouse model used in the current study does not typically progress to plaque rupture, which is a recognised limitation of the *ApoE^−/−^* model [64]. Therefore, future investigations should examine the role of Hpse in plaque rupture using models that better reflect rupture-prone plaque phenotypes.

Currently, statins are the most widely used lipid-lowering drugs for the clinical management of atherosclerotic cardiovascular disease [55]. In addition to their lipid-lowering effects, statins are also known to have anti-inflammatory effects [65]. Given that Hpse has been implicated in promoting various inflammatory processes, it is plausible that statins may indirectly influence Hpse activity through their anti-inflammatory effects. However, to date, there are no reports of statins or other lipid-lowering therapies regulating Hpse expression or activity in the context of atherosclerosis or diabetes-accelerated atherosclerosis. Therefore, it would be of interest to investigate whether lipid-lowering agents, such as statins, modulate Hpse expression and activity under these disease conditions.

In conclusion, the present work discovers Hpse as a critical proinflammatory factor underlying the diabetes-accelerated development of advanced plaques with a more unstable phenotype. This study therefore identifies Hpse as a potential new drug target for the clinical treatment of atherosclerotic cardiovascular disease in patients with diabetes, and prompts further in-depth studies into Hpse’s mechanism of action.

Building on the findings from the present work, future investigations should be expanded on several key directions. First, the role of Hpse in plaque rupture should be investigated using established rupture-prone models, such as tandem stenosis or transverse aortic constriction, particularly under diabetic conditions [66]. Second, the mechanistic actions of Hpse in atherosclerotic cardiovascular disease, both in the presence and absence of diabetes, should be further elucidated using either genetic or pharmacological approaches to block/inhibit Hpse expression and activity. As suggested above, these studies should be complemented by advanced technologies, such as single-cell RNA sequencing, multi-parameter flow cytometry, or spatial transcriptomics, to define the molecular pathways underlying Hpse-mediated effects. Furthermore, evaluating the efficacy of Hpse inhibitors at different stages of atherosclerosis, including diabetes-accelerated disease, will be critical to determine their therapeutic potential. The potential additive or synergistic effects of combining Hpse inhibition with established lipid-lowering therapies, such as statins, should also be explored. Collectively, these studies will help to further direct the development of Hpse-targeted therapeutic strategies in atherosclerotic cardiovascular disease and its associated complications.

## Clinical perspectives

- Hpse, a pro-inflammatory enzyme responsible for the cleavage of HSs in the endothelial glycocalyx and subendothelial ECM is implicated in the development of atherosclerosis and diabetes, but no study has specifically addressed the role of Hpse in diabetes-accelerated atherosclerosis.
- Using a novel transgenic mouse model, we found that *Hpse* gene deficiency significantly reduces the diabetes-accelerated development of atherosclerosis in STZ-treated diabetic *ApoE^−/−^* mice independent of changes to blood glucose and lipid levels. In addition, Hpse deficiency afforded the formation of atherosclerotic lesions with a more stable phenotype in diabetic *ApoE^−/−^* mice that exhibited increased collagen and SMC content and reduced necrotic core size.
- The present study indicates that Hpse is a potential new therapeutic target for protecting against the development of unstable atherosclerotic lesions in patients with diabetes.

## Supplementary Material

Supplementary Figures S1-S6

## Data Availability

The datasets used and/or analysed during the current study are available from the corresponding author on reasonable request.

## Open Access

Open access for this article was enabled by the participation of La Trobe University in an all-inclusive Read & Publish agreement with Portland Press and the Biochemical Society under an agreement with CAUL.

## Abbreviations

ANOVA: Analysis of variance
ApoE: Apolipoprotein E
CBA: Cytometric bead array
ECM: Extracellular matrix
H&E: Haematoxylin/eosin
Hpse: Heparanase
HS: Heparan sulfate
IHC: Immunohistochemical
IL-1β: Interleukin-1 beta
IL-6: Interleukin-6
IP: Intraperitoneal
MCP-1: Monocyte chemoattractant protein-1
MMP: Matrix metalloproteinase
PSR: Picrosirius red/fast green
SMC: Smooth muscle cell
STZ: Streptozotocin
TNF-α: Tumor necrosis factor-alpha
TLR: Toll-like receptor
