# Supplementary material for: Heparanase gene deficiency suppresses atherosclerosis progression and enhances plaque stability in apolipoprotein E gene knockout mice with diabetes
Source: Clin Sci (Lond). 2026 Jul 10;140(8):1599–615. doi: 10.1042/CS20250817 (PMC13358729; doi:10.1042/CS20250817)
Supplement: Supplementary Figures S1-S6 [file CS-2025-0817_supp.pdf]

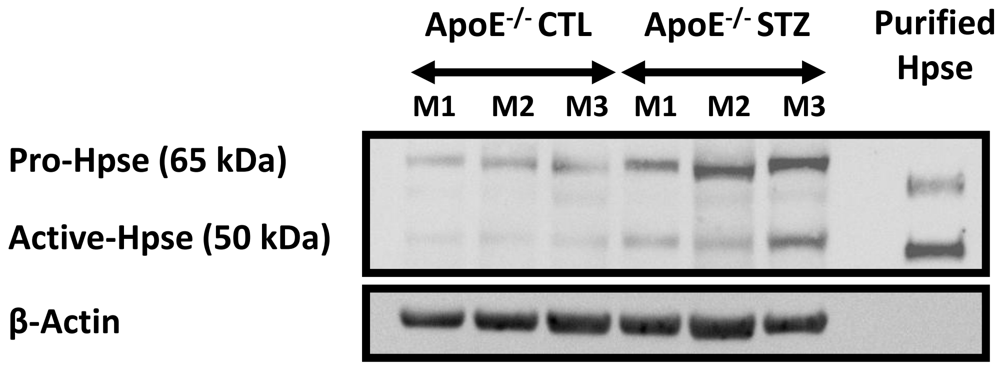

Figure S1. Hpse expression is increased in the spleen of STZ-treated ApoE$^{-/-}$ mice. Expression of Hpse protein in the spleen of mice was detected by Western blotting. Spleen tissue lysates (30 µg) and purified Hpse positive control (30 ng) were probed with anti-Hpse antibody (Abcam 85543; 1:1000). β-Actin was used as a loading control. M: Mouse (3 mice per group).

Original and uncropped images of Western blots

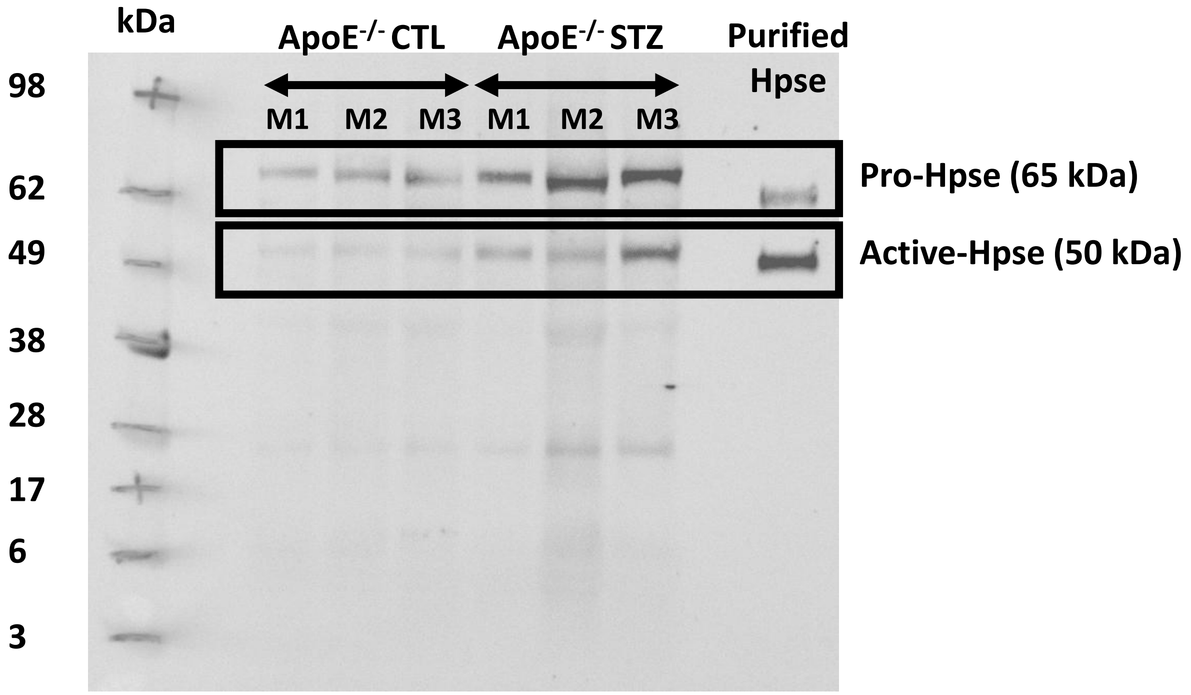

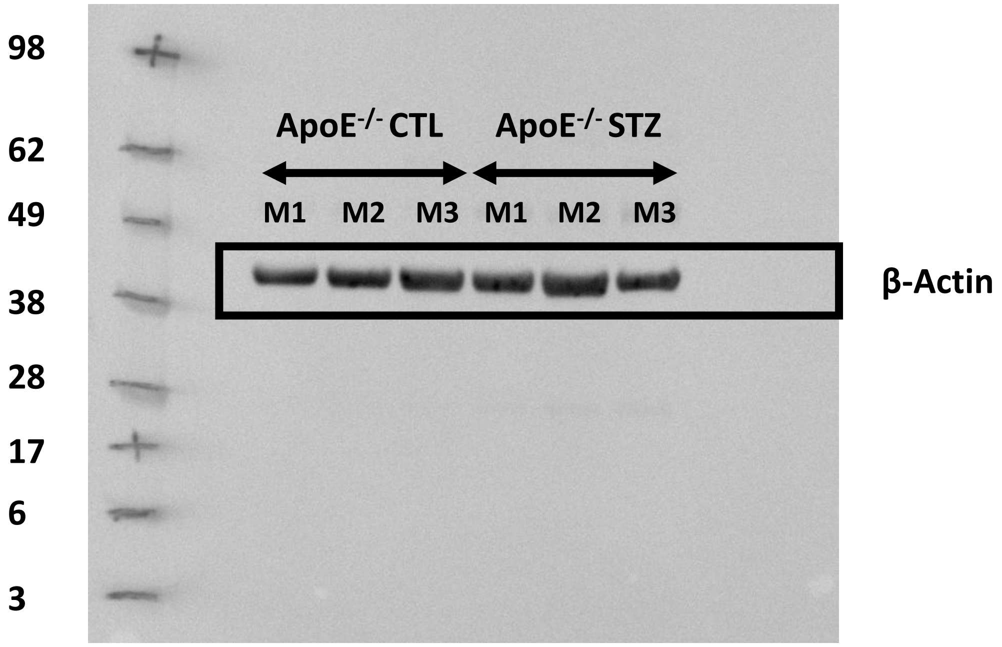

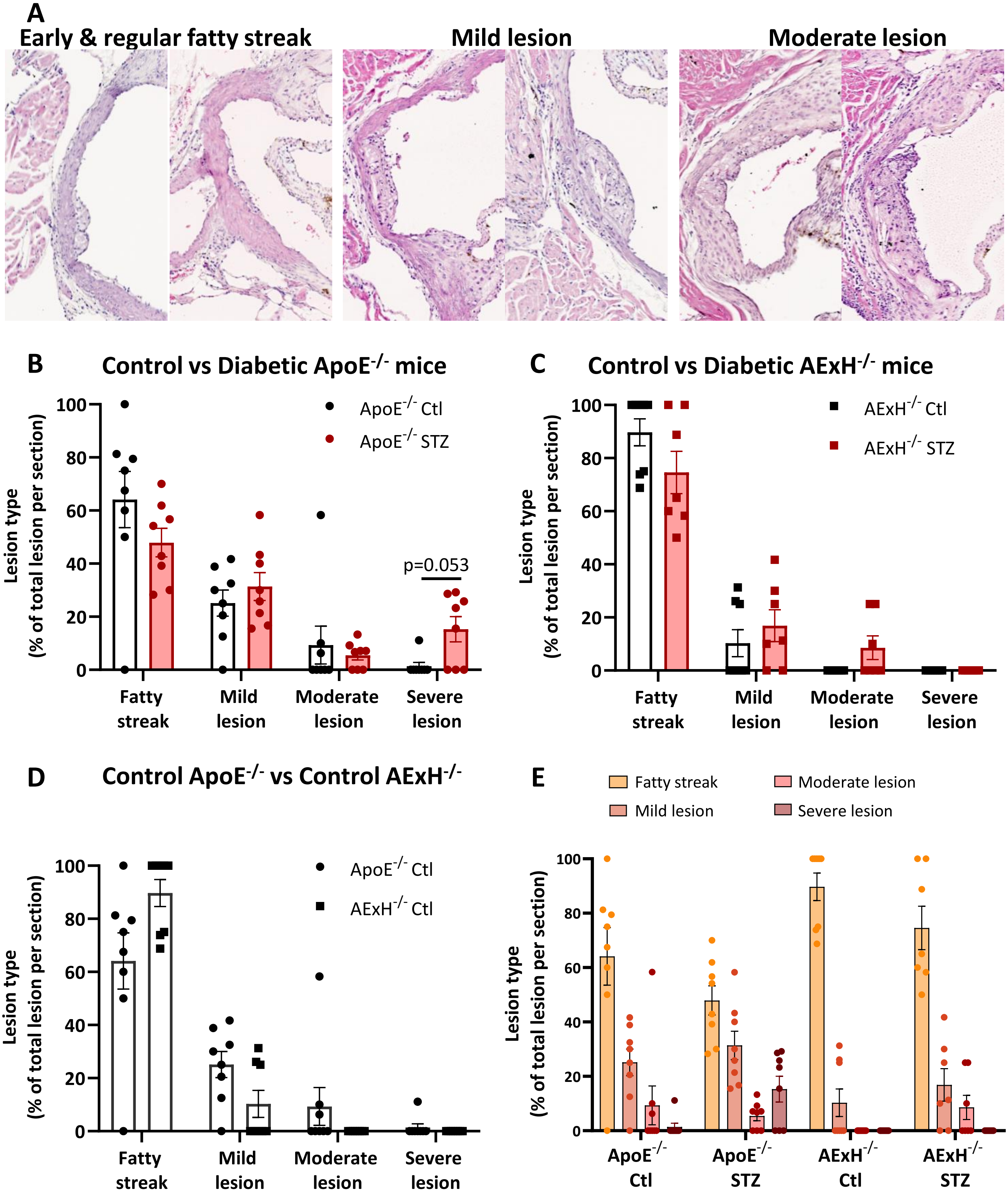

Figure S2. Classification of atherosclerotic lesions developed in the aortic sinus of mice.

(A) Representative images of different atherosclerotic lesion types in H&E-stained aortic sinus tissue from STZ-treated, diabetic AExH$^{-/-}$ mice. Quantification and comparison of atherosclerotic lesion type in (B) control and diabetic ApoE$^{-/-}$ mice only, (C) control and diabetic AExH$^{-/-}$ mice, and (D) control groups only. (E) Atherosclerotic lesion types developed in aortic sinus of all mouse groups. Data represent the mean ± SEM (n=7–8). Statistically significant differences determined by multiple t-test, followed by the Holm-Sidak method (* $P < 0.05$, ** $P < 0.01$)

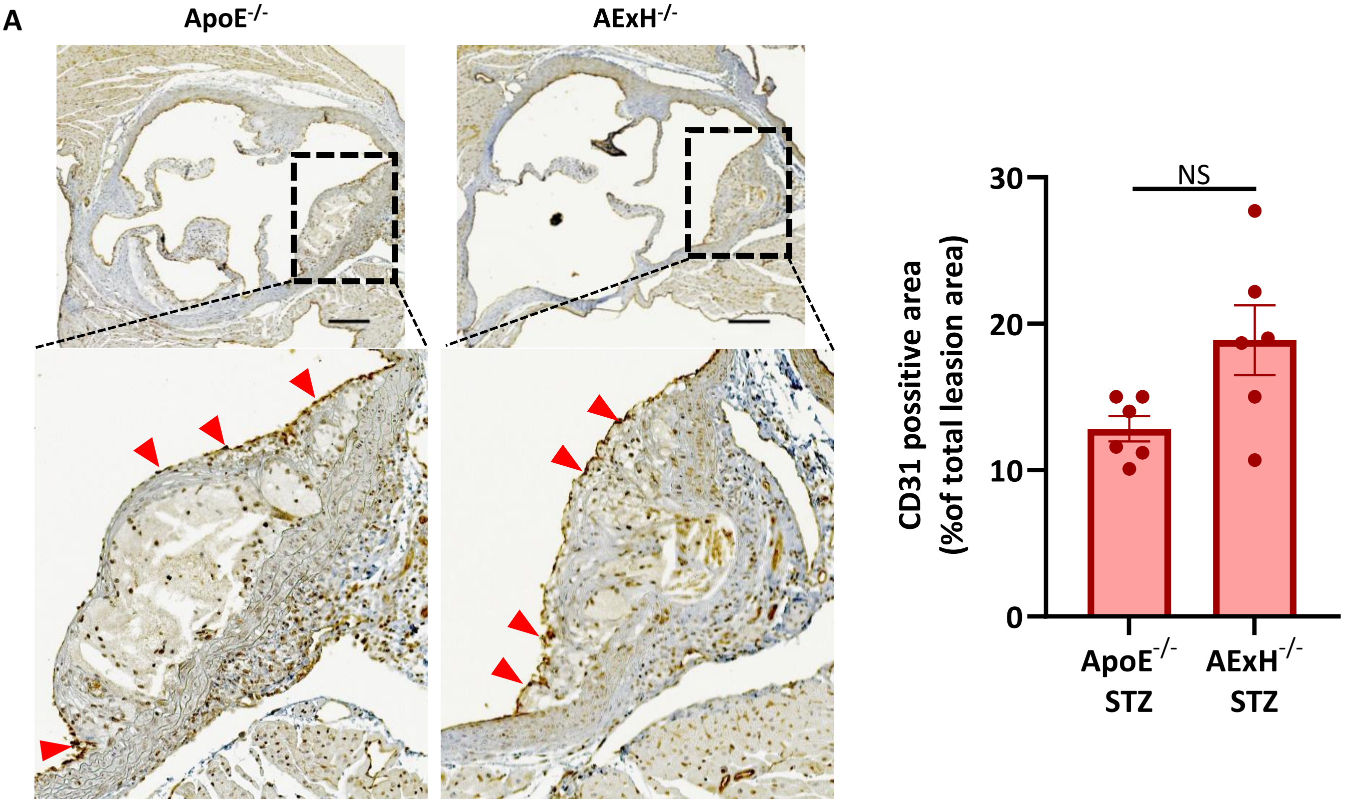

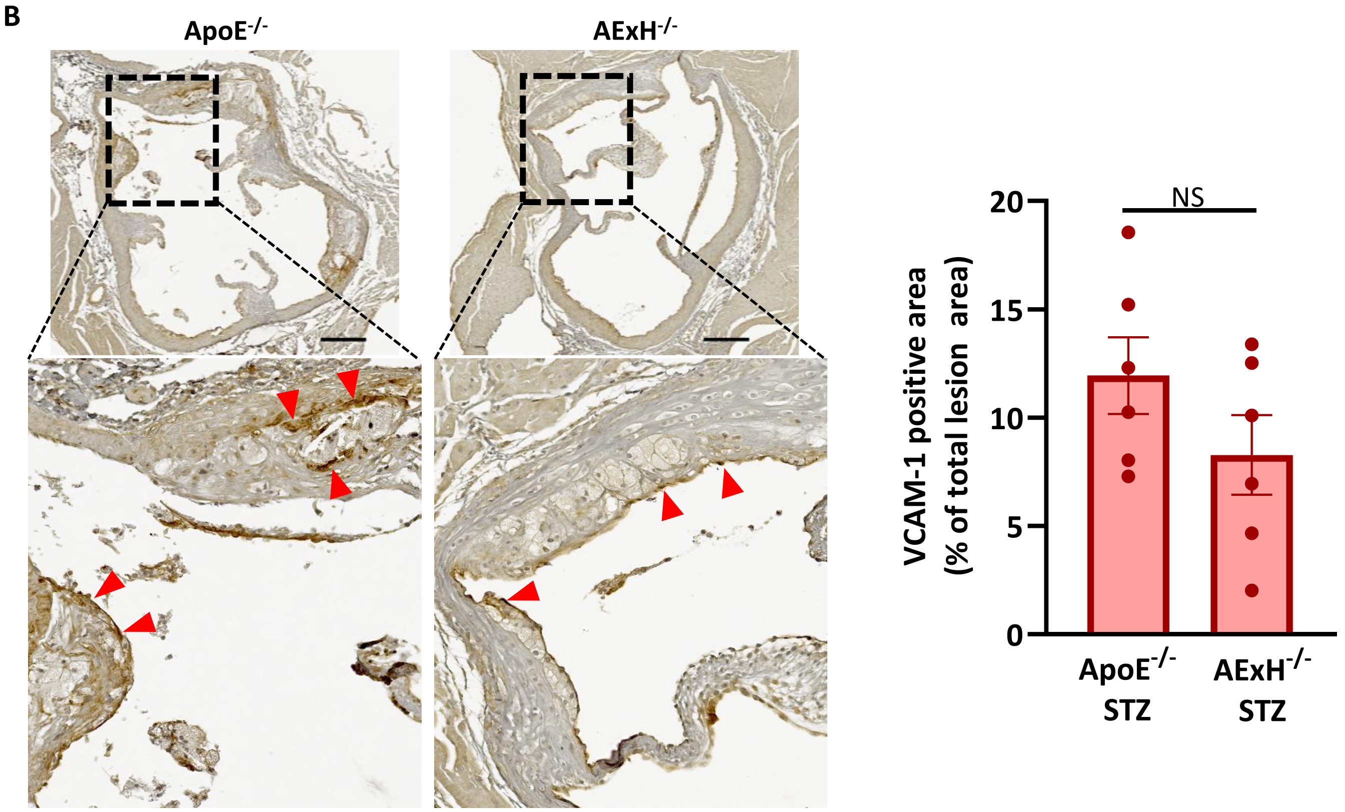

Figure S3. Hpse deficiency does not affect endothelial cell content and activation within atherosclerotic lesion during diabetes.

Representative images of IHC-stained aortic sinus atherosclerotic lesions from STZ-treated diabetic ApoE$^{-/-}$ and AExH$^{-/-}$ mice and quantification of (A) endothelial cell content (CD31) and (B) local VCAM-1 expression in atherosclerotic lesions. Scale bars indicate 200 µm. Data represents the mean ± SEM (n=6). Statistically significant differences determined by two-tailed Mann-Whitney test (NS: not significant).

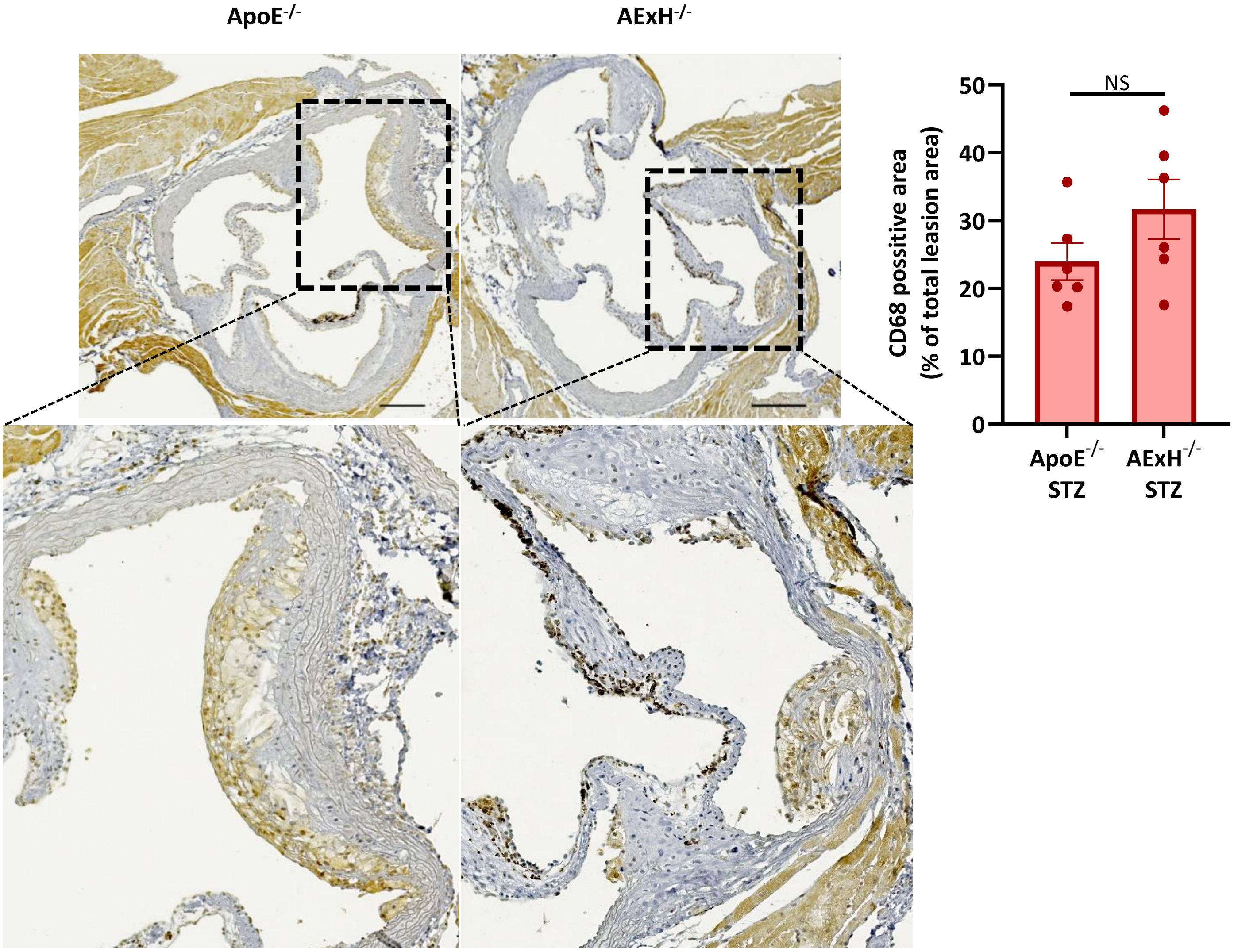

Figure S4. Hpse deficiency does not affect macrophage content within atherosclerotic lesion during diabetes. Representative images of IHC-stained aortic sinus atherosclerotic lesions from STZ-treated, diabetic ApoE$^{-/-}$ and AExH$^{-/-}$ mice and quantification of CD68 expression. Scale bars indicate 200 µm. Data represents the mean ± SEM (n=6). Statistically significant differences determined by two-tailed Mann-Whitney test (NS: not significant).

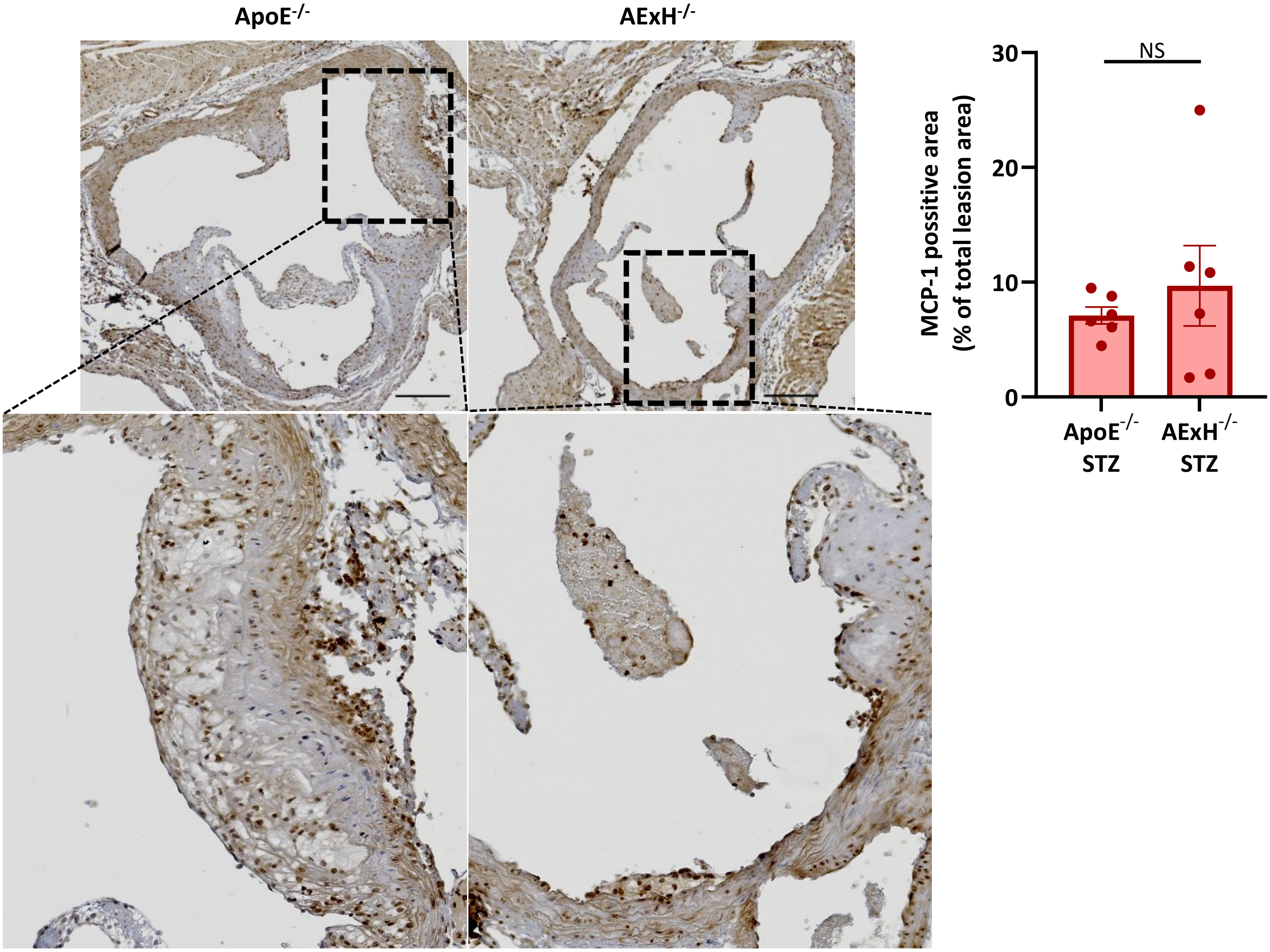

Figure S5. Hpse deficiency does not affect the local expression of MCP-1 within atherosclerotic lesion during diabetes.

Representative images of IHC-stained aortic sinus atherosclerotic lesions from STZ-treated, diabetic ApoE$^{-/-}$ and AExH$^{-/-}$ mice and quantification of MCP-1 expression. Scale bars indicate 200 µm. Data represents the mean ± SEM (n=6). Statistically significant differences determined by two-tailed Mann-Whitney test (NS: not significant).

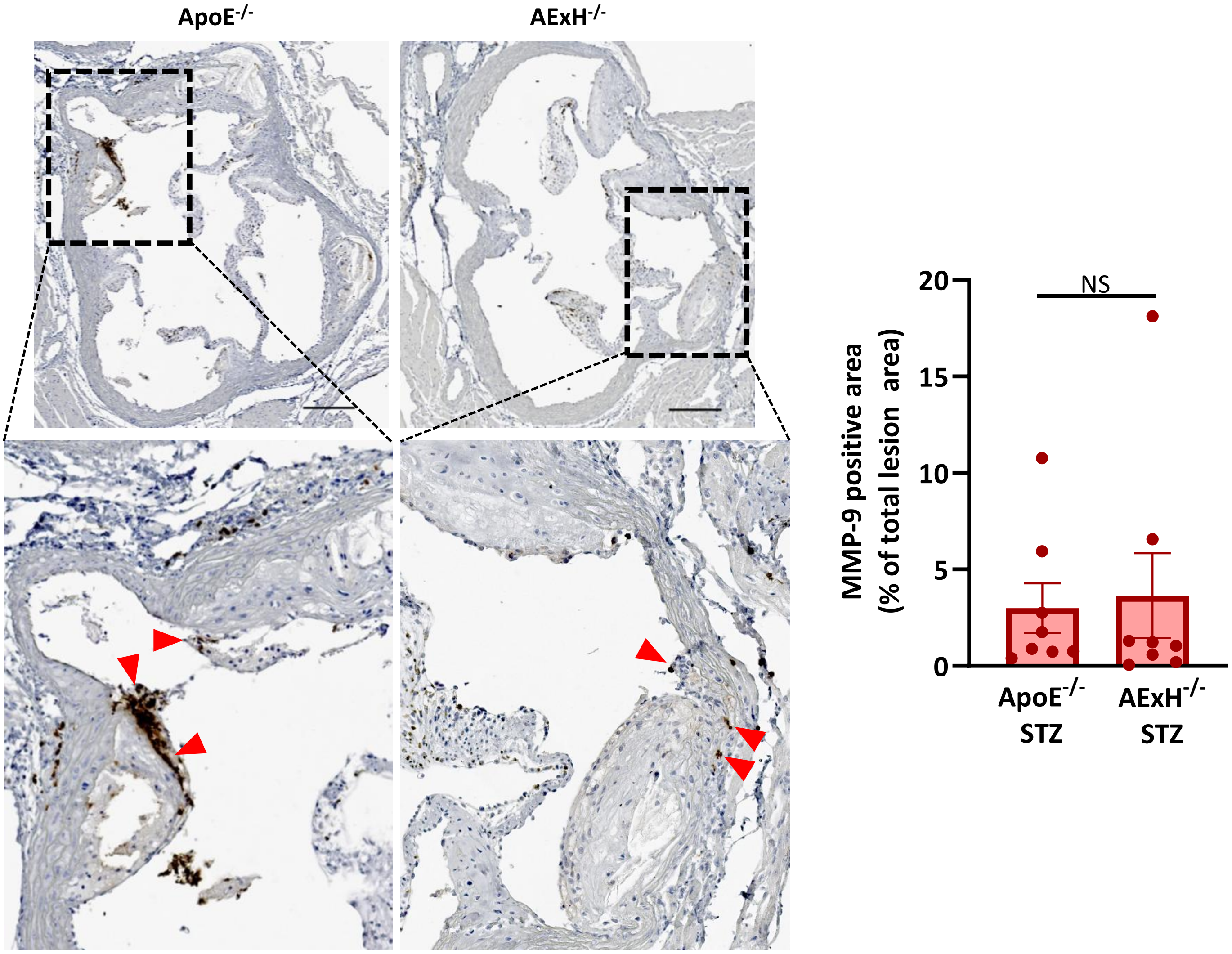

Figure S6. Hpse deficiency does not affect the local expression of MMP9 within atherosclerotic lesion during diabetes.

Representative images of IHC-stained aortic sinus atherosclerotic lesions from STZ-treated, diabetic ApoE$^{-/-}$ and AExH$^{-/-}$ mice and quantification of local expression of MMP9 in atherosclerotic lesions of mice. Scale bars indicate 200 µm. Data represents the mean ± SEM (n=8). Statistically significant differences determined by two-tailed Mann-Whitney test (NS: not significant).
